# MiR-146a-5p delivered by hucMSC extracellular vesicles modulates the inflammatory response to sulfur mustard-induced acute lung injury

**DOI:** 10.1186/s13287-023-03375-8

**Published:** 2023-05-30

**Authors:** Zhipeng Pei, Jinfeng Cen, Xinkang Zhang, Chuchu Gong, Mingxue Sun, Wenqi Meng, Guanchao Mao, Jingjing Wan, Bingyue Hu, Xiaowen He, Qingqiang Xu, Hua Han, Kai Xiao

**Affiliations:** 1 Department of Protective Medicine Against Chemical Agents, Faculty of Naval Medicine, Naval Medical University, Shanghai, 200433 China; 2 Department of Clinical Pharmacy, School of Pharmacy, Naval Medical University, Shanghai, 200433 China; 3 School of Traditional Chinese Materia Medica, Shenyang Pharmaceutical University, Shenyang, 110016 China; 4 Origincell Technology Group Co., Ltd., Shanghai, 201203 China; 5 School of Medicine, Tongji University, Shanghai, 200092 China

**Keywords:** hucMSC-EVs, Sulfur mustard, Inflammation, miR-146a-5p, TRAF6

## Abstract

**Background:**

Sulfur mustard (SM) is a highly toxic chemical warfare agent that has caused numerous casualties during wars and conflicts in the past century. Specific antidotes or therapeutic strategies are rare due to the complicated mechanism of toxicity, which still awaits elucidation. Clinical data show that acute lung injury (ALI) is responsible for most mortality and morbidity after SM exposure. Extracellular vesicles are natural materials that participate in intercellular communication by delivering various substances and can be modified. In this study, we aim to show that extracellular vesicles derived from human umbilical cord mesenchymal stromal cells (hucMSC-EVs) could exert therapeutic effects on SM-induced ALI, and to explain the underlying mechanism of effects.

**Methods:**

MiR-146a-5p contained in hucMSC-EVs may be involved in the process of hucMSC-EVs modulating the inflammatory response to SM-induced ALI. We utilized miR-146a-5p delivered by extracellular vesicles and further modified hucMSCs with a miR-146a-5p mimic or inhibitor to collect miR-146a-5p-overexpressing extracellular vesicles (miR-146a-5p^+^-EVs) or miR-146a-5p-underexpressing extracellular vesicles (miR-146a-5p^−^-EVs), respectively. Through in vivo and in vitro experiments, we investigated the mechanism.

**Results:**

The effect of miR-146a-5p^+^-EVs on improving the inflammatory reaction tied to SM injury was better than that of hucMSC-EVs. We demonstrated that miR-146a-5p delivered by hucMSC-EVs targeted TRAF6 to negatively regulate inflammation in SM-induced ALI models in vitro and in vivo.

**Conclusion:**

In summary, miR-146a-5p delivered by hucMSC-EVs targeted TRAF6, causing hucMSC-EVs to exert anti-inflammatory effects in SM-induced ALI; thus, hucMSC-EVs treatment may be a promising clinical therapeutic after SM exposure.

**Graphical Abstract:**

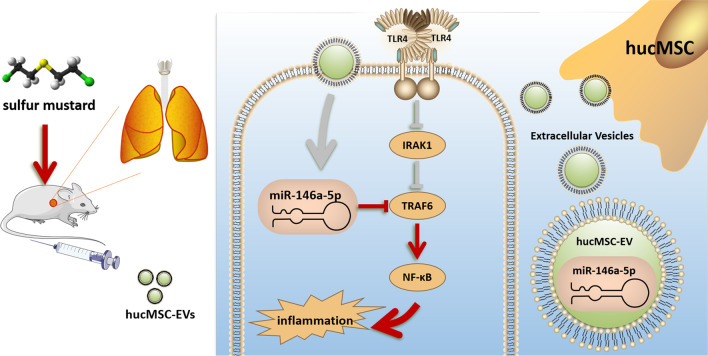

**Supplementary Information:**

The online version contains supplementary material available at 10.1186/s13287-023-03375-8.

## Background

With its characteristic simple structure, easy availability and convenience in creating stockpiles, sulfur mustard (2,2-dichlorodiethyl sulfide, SM) is one of the chemical warfare agents most likely to be used in battlefields as well as in terrorist attacks. Although it has been listed on Schedule I of the Chemical Weapons Convention, it was still used in the conflicts in Syria recently and caused numerous injuries and casualties [1–3]. The accidental leakage of SM poses a threat to the environment and public health, as it is one of the main chemical warfare agents of Japan’s abandoned chemical weapons (ACWs) in China [4, 5]. According to clinical data on SM victims, acute lung injury (ALI) is responsible for the majority of the mortality and morbidity after SM exposure [6–8]. Nevertheless, there are no effective antidotes for treating SM-induced lung injury. The common therapeutic strategy is palliative care with life-saving interventions during the acute phase and symptomatic treatment during the chronic phase, but the effects often fall short of expectations [9, 10]. Therefore, improved approaches to treating SM-induced ALI remain a critical goal of SM research.

In recent years, because no antidote is available for treating SM poisoning, various biological products and cell transplantation have been developed for the purpose of SM poisoning treatment [11]. During the past few years, widespread attention has been given to stem cell therapy due to its great therapeutic potential, especially using mesenchymal stem cells (MSCs) and their extracellular vesicles [12, 13]. We previously found that bone marrow mesenchymal stem cells (BMSCs) and their exosomes derived from mice have protective effects against lung injury induced by SM [14, 15]. However, human umbilical cord MSCs (hucMSCs) have the advantages of being less ethically controversial, having abundant tissue sources, and having low immunogenicity compared with BMSCs from mice [16, 17]. HucMSCs have become the dominant type of stem cell used for allogeneic cell therapy and may be more suitable for the clinical treatment of SM-induced lung injury. HucMSC extracellular vesicles (hucMSC-EVs) are microvesicles secreted from hucMSCs into the extracellular environment [18]. Extracellular vesicles contain various proteins, microRNAs (miRNAs) and mRNAs that can be transmitted to target cells and participate in cell-to-cell communication [19]. Given the growing evidence that hucMSCs primarily rely on the release of extracellular vesicles to demonstrate therapeutic effects, extracellular vesicles may be a better choice for treating SM poisoning by avoiding the administration of live cells, which arouses concern related to tumor formation and long-term safety [20]. In addition, the treatment ability of the extracellular vesicles could be further improved by modifying hucMSCs [21].

In the present paper, we researched the therapeutic quality of hucMSC-EVs on ALI induced by SM and explored the mechanism of action. HucMSC-EVs exhibited strong therapeutic effects, as revealed by the improved survival rate and functional indicators, especially the reduced pulmonary inflammatory response of mice after SM injury. Furthermore, we examined the anti-inflammatory effects and mechanisms in SM-induced lung injury in vitro and in vivo. We found that hucMSC-EVs exerted anti-inflammatory effects in SM-induced ALI at least partly through miR-146a-5p delivered by hucMSC-EVs, which targeted TRAF6.

## Methods

### Animals and cells

All mouse studies complied with the United States National Research Council’s Guide for the Care and Use of Laboratory Animals and the Committee on Ethics of Medical Research of the Naval Medical University’s (Approval no.20180308007, Shanghai, China) policies governing the humane and ethical treatment of the experimental subjects. ICR mice (8 weeks old) were obtained from the Naval Medical University’s animal center. All the mice lived under a 12/12-h light/dark cycle environment with a controlled temperature of 22 ± 2 °C. They had free access to adequate food and clean water.

Mouse macrophages (RAW264.7 cells), human lung epithelial cells (BEAS-2B cells) and human lung fibroblasts (HFL-1 cells) were purchased from the National Collection of Authenticated Cell Cultures (Shanghai, China). The culture medium for both RAW264.7 cells and BEAS-2B cells was Dulbecco's modified Eagle medium (DMEM) (HyClone, USA) with 10% fetal bovine serum (FBS) (Gibco, USA) and 100 μg/mL penicillin–streptomycin (HyClone, USA). The hucMSCs were donated to our research group by Origincell Technology Group Company and were cultured in mesenchymal stem cell basal medium (Dakewe, China) containing 5% cell culture supplements (animal serum-free, xenogeneic-free, EliteCell, USA). Alpha minimum essential medium (α-MEM) supplemented with 10% FBS and 100 μg/mL penicillin–streptomycin was used to culture HFL-1 cells. All the cells were grown at 37 °C under 5% CO_2_ in an incubator.

### Extracellular vesicles separation

Cell supernatants were employed to isolate and purify extracellular vesicles. The cells were cultured with animal serum-free, xenogeneic-free cell culture supplements instead of the usual FBS to prevent interference from serum extracellular vesicles. To remove any dead cells and cell debris, the supernatants of the cells were centrifuged at 300 × *g* for 10 min, 2000 × *g* for 10 min at 4 °C and then passed through a 0.22 μm filter (Millipore, USA). After centrifugation at 10,000 × *g* for 30 min at 4 °C, the supernatants were ultracentrifuged at 100,000 × *g* for 70 min using the ultracentrifuge (Optima XPN-80, SW32 rotor, Beckman Coulter, USA) at 4 °C. The supernatants were removed, and phosphate buffered saline (PBS) (HyClone, USA) was added to the pellets for washing by ultracentrifugation at 100,000 × *g* for another 70 min at 4 °C. The pellets contained the extracellular vesicles required for the experiments. The final pellets were diluted with 200 μL of PBS and stored at − 80 °C until further use.

### Extracellular vesicles characterization

The purified extracellular vesicles were suspended in phosphoric acid buffer. The samples were dropped onto carbon-coated copper grids, adsorbed for 90 s and then stained with uranium acetate solution for 30 s. Transmission electron microscopy was adopted to observe the morphology of the extracellular vesicles. The size distribution of the extracellular vesicles was assessed by nanoparticle tracking analysis. CD9 (1:1000, 13174, CST, USA), CD63 (1:1000, 25682-1-AP, Proteintech, China), CD81 (1:1000, SAB3500454, Sigma, USA) and Cav-1 (1:1000, ab2910, Abcam, UK) are extracellular vesicles-specific biomarkers, and their expression can be visualized by western blotting. Extracellular vesicles labeled with Dil fluorescent dye (Invitrogen, USA) and excess fluorescent dye was removed by ultracentrifugation at 100,000 × *g* for 1 h then washed twice. The labeled extracellular vesicles were incubated with the cells and the cell fluorescence was observed by confocal microscopy to judge whether the extracellular vesicles could be ingested by the target cells.

### Mouse model and hucMSC-EVs administration

The ALI mouse model was established by subcutaneous injection of SM solution. According to previous studies, a subcutaneous injection of SM can cause ALI, and the exposure dose can be accurately controlled in a safe way [14, 15, 22]. In brief, SM diluted to the preset concentration (40 or 30 mg/kg weight used to calculate survival curve or evaluate other experimental indicators) with propylene glycol (Sigma, USA) was subcutaneously injected into the middle of the skin on the back of the mouse. ALI mice were randomly divided into different treatment groups: the hucMSC-EV group, HFL-1-EV group, miR-146a-5p^+^ hucMSC-EV group, miR-146a-5p^−^ hucMSC-EV group and N-acetyl cysteine (NAC) group. In our study, NAC was a positive control because it has been reported as a leading candidate for the therapy of SM-induced pulmonary toxicity. HFL-1-EVs were used as the negative control. Mice in different types of extracellular vesicles groups were treated with 3 × 10^8^ particle extracellular vesicles suspended in 150 μl of PBS via tail vein injection on the first and third days after SM exposure. Mice in the NAC group were intragastrically administered NAC (200 mg/kg) (Sigma, USA) once a day. At the end of the experiment, mice were injected intraperitoneally with pentobarbital sodium (1% v/v) for anesthesia and then experimental materials were collected. Other mice were euthanized by CO_2_ asphyxiation. The procedures were approved and performed as indicated by Committee on Ethics of Medical Research, Naval Medical University.

### Bronchoalveolar lavage and the lung wet/dry weight ratio

The mice were deeply anesthetized before the thoracic cavity was opened and the trachea was exposed. The trachea was secured with tweezers, and a small gap was cut with scissors. A tube was inserted into the trachea through the gap, through which bronchoalveolar lavage fluid (BALF) was collected by carefully and slowly instilling and withdrawing PBS (0.5 ml) into the lung. After centrifugation (3000 rpm, 10 min), the protein concentration in the BALF was detected by the bicinchoninic acid assay (BCA) (Thermo Scientific, USA) method. To compare lung water contents, mice were sacrificed, and the wet lung tissue weight was obtained by weighing. Then, the tissues were dehydrated for 72 h in a drying oven at 65 °C and weighed again to obtain the dry weight. The ratio of the wet weight to dry weight was the lung wet/dry weight ratio.

### Histopathologic examination

After deep anesthesia, the thoracic cavity of mice was opened by median sternotomy. The blood was collected via the left ventricle using a 25 gauge needle. Blood was withdrawn slowly to prevent the heart from collapsing. The lungs were flushed with 10 ml physiological saline and then immediately removed. One of the lungs was used for histopathologic examination and immunohistochemistry. The others were rinsed with saline and used for other experiments. Paraformaldehyde solution (4%, v/v) was used to fix lung tissues. After 24 h, lung tissues were embedded in paraffin. To perform histopathologic examination, the embedded tissue that was sectioned was stained with hematoxylin–eosin (H&E). Tissue sections were investigated, and pictures were taken using a light microscope. Following the recommendations of the American Thoracic Society Official workshop report [23, 24], all tissue sections were evaluated in a blinded manner by two pathologists using five pathological indexes of neutrophils in the alveolar space, neutrophils in the interstitial space, hyaline membranes, proteinaceous debris filling the airspaces and alveolar septal thickening.

### Immunohistochemical analysis

Immunohistochemical analysis was used to detect the expression of NF-κB and TRAF6. After deparaffinization and rehydration, the sections were placed in 3% hydrogen peroxide for 15 min to quench endogenous peroxidases. Then, 0.01 M citrate buffer was employed to retrieve the antigens. After blocking with 3% BSA (Sigma, USA) for 30 min, the sections were incubated with the NF-κB (1:400, 6956, CST, USA) or TRAF6 (1:200, 66498-1-Ig, Proteintech, China) primary antibody at 4 °C for more than 12 h and were then stained using diaminobenzidine reagent.

### Quantitative real-time PCR

RNAiso Plus reagent (Takara, Japan) was used to extract total RNA from tissues or cell samples. Total RNA (DNA-free) was reverse transcribed with HiScript III qRT SuperMix (Vazyme, China). Quantitative real-time PCR analysis was performed using SYBR green master mix (Vazyme, China). All reactions were performed in triplicate. The relative expression level of target genes was normalized to that of endogenous reference control GAPDH. Specific primers and the transcribed cDNA were used for PCR amplification. TLR4 forward sequence (5′–3′): ATGGCATGGCTTACACCACC, reverse sequence (5′–3′): GAGGCCAATTTTGTCTCCACA; TRAF6 forward sequence (5′–3′): AAAGCGAGAGATTCTTTCCCTG, reverse sequence (5′–3′): ACTGGGGACAATTCACTAGAGC; IRAK1 forward sequence (5′–3′): CCACCCTGGGTTATGTGCC, reverse sequence (5′–3′): GAGGATGTGAACGAGGTCAGC; TNF-α forward sequence (5′–3′): CCCTACCACTCAGATCATCTTCT, reverse sequence (5′–3′): GCTACGACGTGGGCTACAG; IL-1β forward sequence (5′–3′): GCAACTGTTCCTGAACTCAACT, reverse sequence (5′–3′): ATCTTTTGGGGTCCGTCAACT; IL-6 forward sequence (5′–3′): TAGTCCTTCCTACCCCAATTTCC, reverse sequence (5′–3′): TTGGTCCTTAGCCACTCCTTC; IL-10 forward sequence (5′–3′): GCTCTTACTGACTGGCATGAG, reverse sequence (5′–3′): CGCAGCTCTAGGAGCATGTG; miR-146a-5p forward sequence (5′–3′): ACACTCCAGCTGGGTGAGAACTGAATTCCA, reverse sequence (5′–3′): TGGTGTCGTGGAGTCG; U6 forward sequence (5′–3′): CTCGCTTCGGCAGCACA, reverse sequence (5′–3′): AACGCTTCACGAATTTGCGT; and GAPDH forward sequence (5′–3′): AACATCTACAAGCCCAACAACAAGG, reverse sequence (5′–3′): GGTTCTGCAATCACATCTTCAAAGTC.

### Western blotting analysis

After washing with precooled PBS, proteins from cells and tissues were lysed and extracted by radio immunoprecipitation assay (RIPA) buffer (Thermo Scientific, USA) mixed with protease inhibitors. The mixtures were centrifuged at 12,000 × *g* for 15 min to collect the supernatant as the protein solution. BCA protein assay kits were used to determine the protein concentration in the solutions. For the western blotting analysis, the protein solution was boiled at 100 °C for 10 min after evenly mixing with 5 × loading buffer. Denatured protein solutions were prepared as previously described [25]. The membranes were incubated with the primary antibodies against TLR4 (1:200, sc-293072, Santa Cruz, USA), TRAF6 (1:1000, 67591, CST, USA), IRAK1 (1:1000, 4504, CST, USA), NF-κB (1:1000, 4764, CST, USA), phospho-NK-κB (1:1000, 3033, CST, USA), β-Actin (1:1000, AF0003, Beyotime, China) at 4 °C for 12 h, followed by incubation with the secondary antibodies at room temperature for 1 h. Then, an enhanced chemiluminescence system (ECL Western Blotting System) (Millipore, USA) was used to visualize the immunoreactive proteins on the membranes. The protein bands were quantified and analyzed with ImageJ 1.34s software.

### Measurement of inflammatory factors with ELISA

Mouse lung tissue was obtained from the mice on the fifth day after SM exposure. The tissue was homogenized and centrifuged at 3500 rpm for 10 min. Cell supernatant samples were collected 24 h after SM exposure. According to the product instructions, the concentrations of cytokines were measured with a multidetection microplate reader using an enzyme-linked immunosorbent assay (ELISA) kit (Westang Bio-tech, China).

### siRNA or miRNA mimic and inhibitor transfection of cells

To overexpress or knockdown the miRNA-146a-5p gene, cells were infected with the miRNA-146a-5p mimic or inhibitor (Thermo Scientific, USA) using Lipofectamine RNAiMAX (Invitrogen, USA). RAW264.7 cells were seeded at a density of 2 × 10^4^ cells/cm^2^ in cell culture plates. After 12 h, cells were transfected with 100 nM siRNA/non-targeting siRNA mixed with Opti-MEM (Invitrogen, USA) and incubated in the cell incubator. After being treated for 36 h, the cells were used for subsequent experiments. HucMSCs were transfected with 100 nM miRNA-146a-5p mimic or inhibitor with Opti-MEM upon reaching 60–70% confluence. After 72 h, the supernatant was collected for extracellular vesicles extraction. The relative expression level of the miRNA-146a-5p gene was compared using quantitative real-time PCR.

### Cell viability assay

A Cell Counting Kit-8 (CCK-8) (Dojindo Laboratories, Japan) was used to detect cell viability. CCK-8 solution was evenly mixed with fresh medium at a ratio of 1:10 according to the product instructions and then added to the cell culture plate. After coculture at 37 °C for 1 h, the absorbance of each well in the culture plate at 450 nm was measured using a microplate reader (BioTek, USA). After deducting blank values, the ratio of the experimental group values to the control group values was used to represent the proliferation ability of the cells.

### Statistical analysis

The experimental data are represented as the mean ± standard deviation of individuals included in each group and representative of at least three independent experiments. SPSS 21.0 software (IBM, USA) was employed for statistical analysis. When there were only two groups in the experiment, significance was determined with the two-tailed Student’s t test (two groups). If there were more than two groups, significance was determined with one-way ANOVA (multiple groups). Statistical significance was defined as *P* < 0.05, which is indicated by one (*) asterisk, and two (**) or three (***) asterisks indicate significance of *P* < 0.01 or *P* < 0.001, respectively.

## Results

### HucMSC-EVs demonstrate therapeutic effects and reduce the inflammatory response in mice after SM injury

**Fig. 1:**
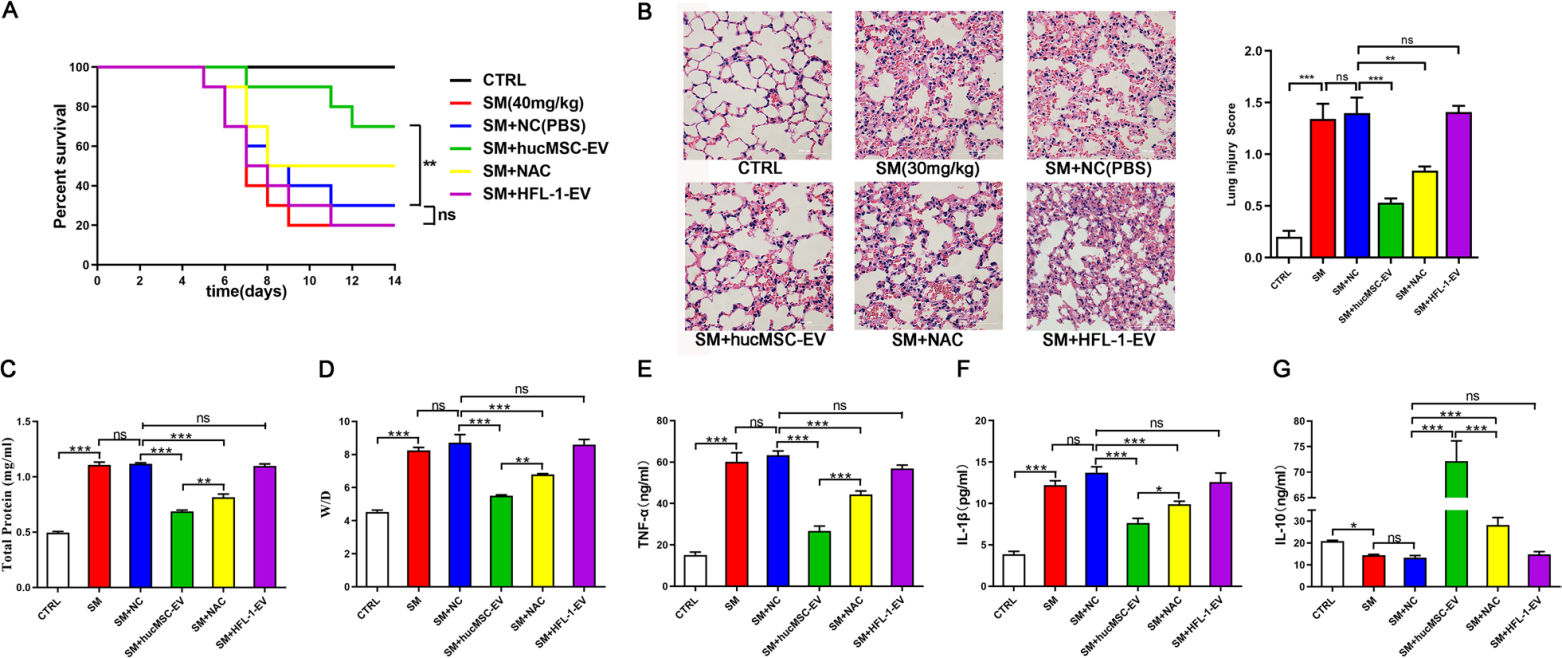
HucMSC-EVs demonstrate therapeutic effects and reduce the inflammatory response in mice after SM injury. **A** Survival curves for the different treatment groups (*n* = 10 mice/group). HucMSC-EV administration significantly improved the survival rate. **B** Representative histological micrograph analysis (× 200, scale bar = 100 μm) and histopathological scores (*n* = 3 mice/group). **C**, **D** Comparison of BALF protein and the wet/dry weight ratio in SM-exposed mice (*n* = 6 mice/group). **E**–**G** ELISA was performed to visualize the expression of the inflammatory factors TNF-α, IL-1β, and IL-10 in mouse lung tissues (*n* = 6 mice/group). The data are presented as the mean ± SD of individuals included in each group and representative of at least three independent experiments. *P < 0.05; **P < 0.01; and ***P < 0.001

The hucMSC that could subcultured in vitro was donated to our research group by Origincell Technology Group Company. Cells were identified based on typical morphological observations with an inverted microscope, phenotypic determination with flow cytometry and multipotent differentiation capacity (Additional file 1: Fig. S1). HucMSC-EVs were isolated from the cell supernatant of passage 3–7 hucMSCs. The identity and purity of the extracellular vesicles were characterized by several methods, such as nanoparticle tracking analysis, transmission electron microscopy, and western blotting (Additional file 1: Fig. S2). To study the therapeutic effects of hucMSC-EVs on mice after SM injury, a stable and optimized mouse model of ALI was established. Detailed information about the general health of the mice in each group was recorded for 14 days, and the survival rate was calculated. At the end of the observation, all mice were euthanized by overdose of anesthetic. Compared to the control group, the dietary intake and physical activity of the mice were significantly reduced twenty-four hours after SM injection. The mice began to lose weight significantly on the third day, and some of them died on the sixth day, which is the reason that subsequent experiments examined tissues from mice after SM injury for 5 days. These SM-induced effects were alleviated by the administration of hucMSC-EVs. HucMSC-EVs administration significantly improved the general health condition and survival rates (Fig. 1A). HFL-1-EVs as the negative control had no effects on the general health and survival rates of the mice.

In the histopathological study, the presence of alveolar septum thickening, pulmonary edema, massive inflammatory cell and exudate infiltration, and hemorrhage after SM injury were confirmed by H&E staining (Fig. 1B). These effects could be improved by hucMSC-EVs injection, while the negative control HFL-1-EVs showed no benefit. In addition, BALF protein (Fig. 1C) and the wet/dry weight ratio (Fig. 1D) were important indicators of the exudation associated with pulmonary edema, confirming that SM injury could be ameliorated by hucMSC-EVs.

ELISA was used to measure inflammatory factors in mouse lung tissues. The levels of TNF-α (Fig. 1E) and IL-1β (Fig. 1F) were markedly augmented after SM injury compared to those in the control group, and their levels were successfully reduced by hucMSC-EVs administration, whereas HFL-1-EVs could not reduce their levels. The level of IL-10 (Fig. 1G), an anti-inflammatory factor, was significantly augmented with hucMSC-EVs treatment. In general, hucMSC-EVs administration markedly reduced the inflammatory response in mice after SM injury.

### MiR-146a-5p delivered by hucMSC-EVs is functionally relevant

**Fig. 2:**
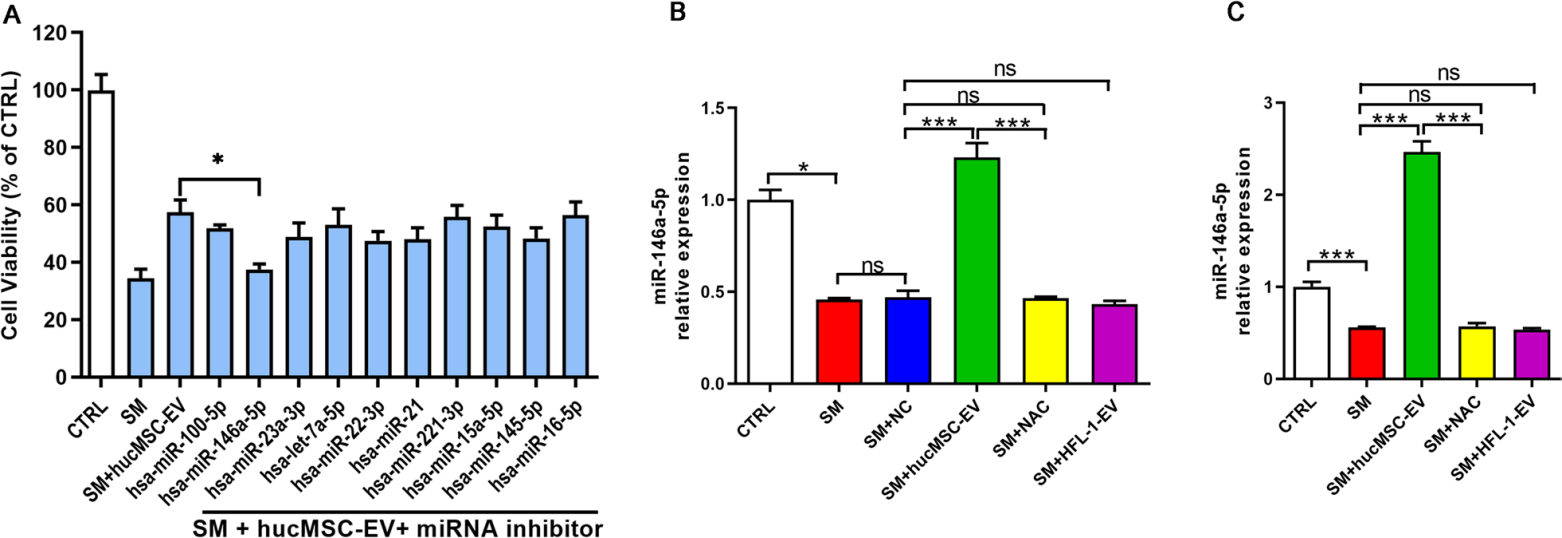
MiR-146a-5p delivered by hucMSC-EVs is functionally relevant. **A** Cell viability of different treatment groups was determined by the CCK-8 assay (*n* = 3). MiR-146a-5p expression in mouse lung tissues (**B**) and BEAS-2B cells (**C**) was visualized by qRT–PCR (*n* = 3). The data are presented as the mean ± SD of individuals included in each group and representative of at least three independent experiments. *P < 0.05; **P < 0.01; and ***P < 0.001

Many studies have reported that miRNAs play an important role in the therapeutic effects of extracellular vesicles, although extracellular vesicles also contain proteins, lipids, metabolites and other components of extracellular vesicles that demonstrate therapeutic effects [26]. We therefore sought to determine the mechanisms by which the miRNAs contained in hucMSC-EVs exert anti-inflammatory effects. According to the functions of some miRNAs already reported and the miRNA components of hucMSC-EVs [19, 27], several miRNA molecules involved in hucMSC-EVs that might be relevant to our study were screened out, including miR-100-5p, miR-146a-5p, miR-23a-3p, let-7a-5p, miR-22-3p, miR-21, miR-221-3p, miR-15a-5p, miR-145-5p and miR-16-5p. We found that the protective effect of hucMSC-EVs on cell viability was significantly reduced when the miR-146a-5p was inhibited, while other miRNA molecules had no significant effects (Fig. 2A).

Many papers have shown that miR-146a-5p is more closely correlated with inflammation [28–30]. Therefore, we believe that miR-146a-5p may be involved in the process by which hucMSC-EVs modulate the inflammatory response to SM-induced ALI. An analysis of miR-146a-5p expression in mouse lung tissues (Fig. 2B) and cells (Fig. 2C) revealed that the levels of miR-146a-5p expression were markedly mitigated after SM injury compared to the control group, and their levels were successfully increased by hucMSC-EVs administration. MiR-146a-5p is an important immunoregulatory miRNA that is reportedly expressed in both MSCs and MSC-derived extracellular vesicles [31]. Therefore, we explored the mechanisms of miR-146a-5p delivered by hucMSC-EVs involvement in the hucMSC-EV-mediated anti-inflammatory effects in SM-induced lung injury.

### MiR-146a-5p negatively regulates SM-induced inflammation

**Fig. 3:**
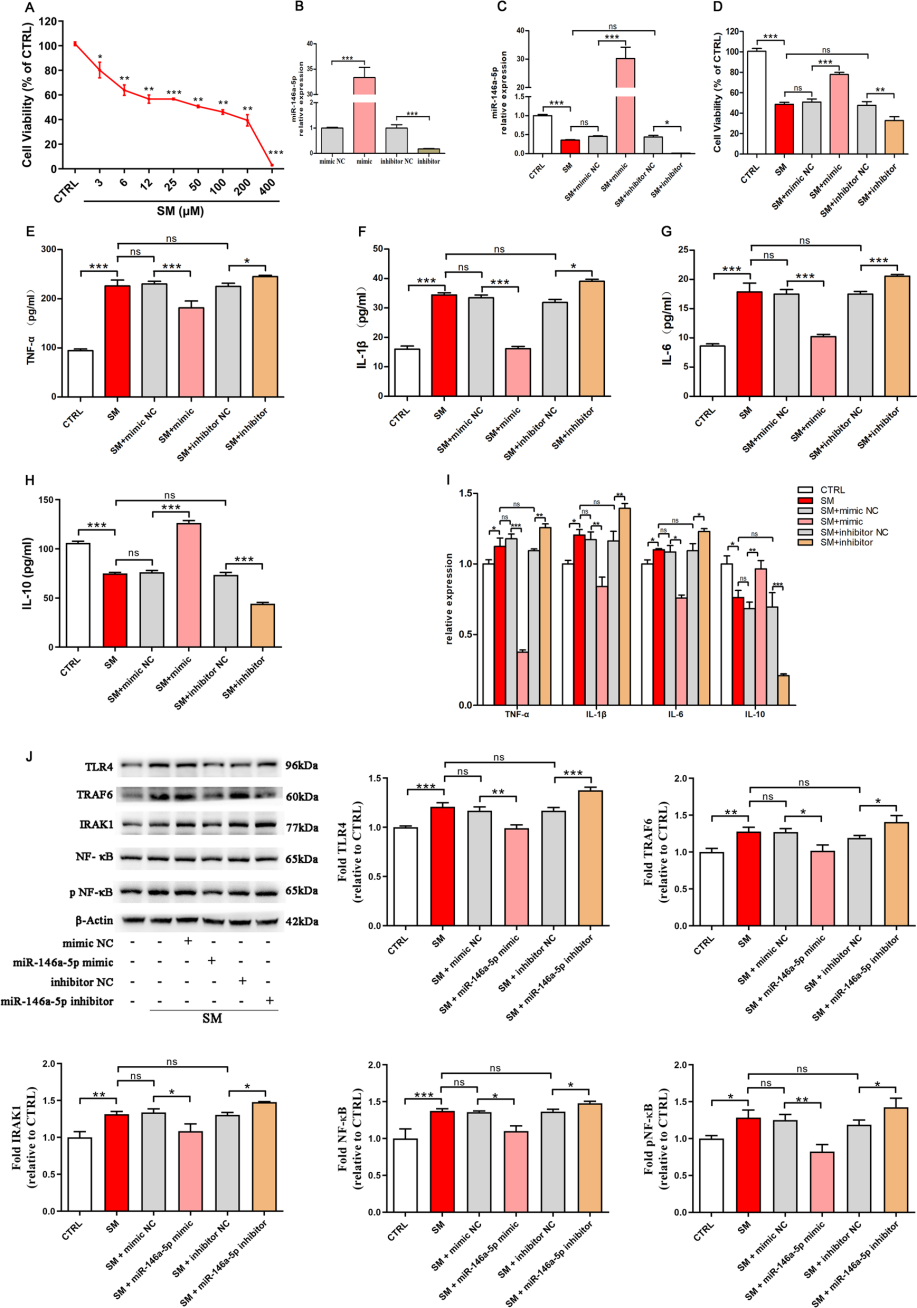
MiR-146a-5p negatively regulates SM-induced inflammation. **A** Untreated or SM-treated RAW264.7 cells were further cultured for 24 h. Cell viability was measured by the CCK-8 assay. The inflammatory cell model was established using RAW264.7 cells treated with SM (50 μM). **B** miR-146a-5p expression change after exogenous addition of its mimic or inhibitor. **C** miR-146a-5p expression in RAW264.7 cells from each group was assessed by qRT–PCR. **D** The cell viability of different treatment groups was determined by the CCK-8 assay. **E**–**H** The levels of TNF-α, IL-1β, IL-6, and IL-10 in the cell supernatant isolated at 24 h after exposure to SM were detected by ELISA. **I** The expression of these factors in the cells was detected by qRT–PCR. **J** The expression of TLR4/NF-κB signaling pathway-related proteins isolated from cells at 24 h after exposure to SM was visualized by western blotting. Full-length blots are presented in Additional file 1: Fig. S5. The data are presented as the mean ± SD of individuals included in each group (*n* = 3) and representative of at least three independent experiments. *P < 0.05; **P < 0.01; and ***P < 0.001

To identify the mechanisms of the anti-inflammatory effects of miR-146a-5p, an inflammatory cell model of SM injury was established using RAW264.7 cells (Fig. 3A), which is a murine lung macrophage cell line widely used in the study of ALI and inflammation [32]. MiR-146a-5p was overexpressed or underexpressed in RAW264.7 cells by transfection of a miR-146a-5p mimic or inhibitor, respectively (Fig. 3B). Following transfection for twelve hours, the cells were exposed to SM for 30 min. As shown in Fig. 3C, miR-146a-5p was significantly downregulated twenty-four hours after SM injury. Cell viability was reduced after SM exposure and was significantly increased or reduced in miR-146a-5p-overexpressing or miR-146a-5p-underexpressing cells, respectively (Fig. 3D), while the negative controls showed no significant effects. These results suggested that miR-146a-5p attenuated SM-induced cytotoxicity and promoted recovery in RAW264.7 cells.

Compared to the NC group, ELISA revealed that the levels of TNF-α (Fig. 3E), IL-1β (Fig. 3F), and IL-6 (Fig. 3G) secreted by RAW264.7 cells were successfully decreased by miR-146a-5p mimic administration, and miR-146a-5p inhibitor administration notably increased their levels after SM injury. The level of the anti-inflammatory factor IL-10 (Fig. 3H) was significantly enhanced with miR-146a-5p mimic treatment. In addition, RT–PCR analysis also showed that the trend in these factors in cells was the same as the ELISA results (Fig. 3I). These results showed that miR-146a-5p significantly suppressed inflammation after SM injury.

One of the most important regulators of inflammation is the TLR4/NF-κB signaling pathway, and we found that in the miR-146a-5p-overexpressing group, the TLR4, TRAF6, IRAK1, NF-κB, and pNF-κB levels all decreased, while those levels were augmented in the miR-146a-5p-underexpressing group compared to those in the NC group (Fig. 3J). In general, miR-146a-5p negatively regulates inflammation induced by SM and is related to the regulation of the TLR4 signaling pathway.

### MiR-146a-5p delivered by hucMSC-EVs targets TRAF6 and mediates the anti-inflammatory effect of hucMSC-EVs in vitro

**Fig. 4:**
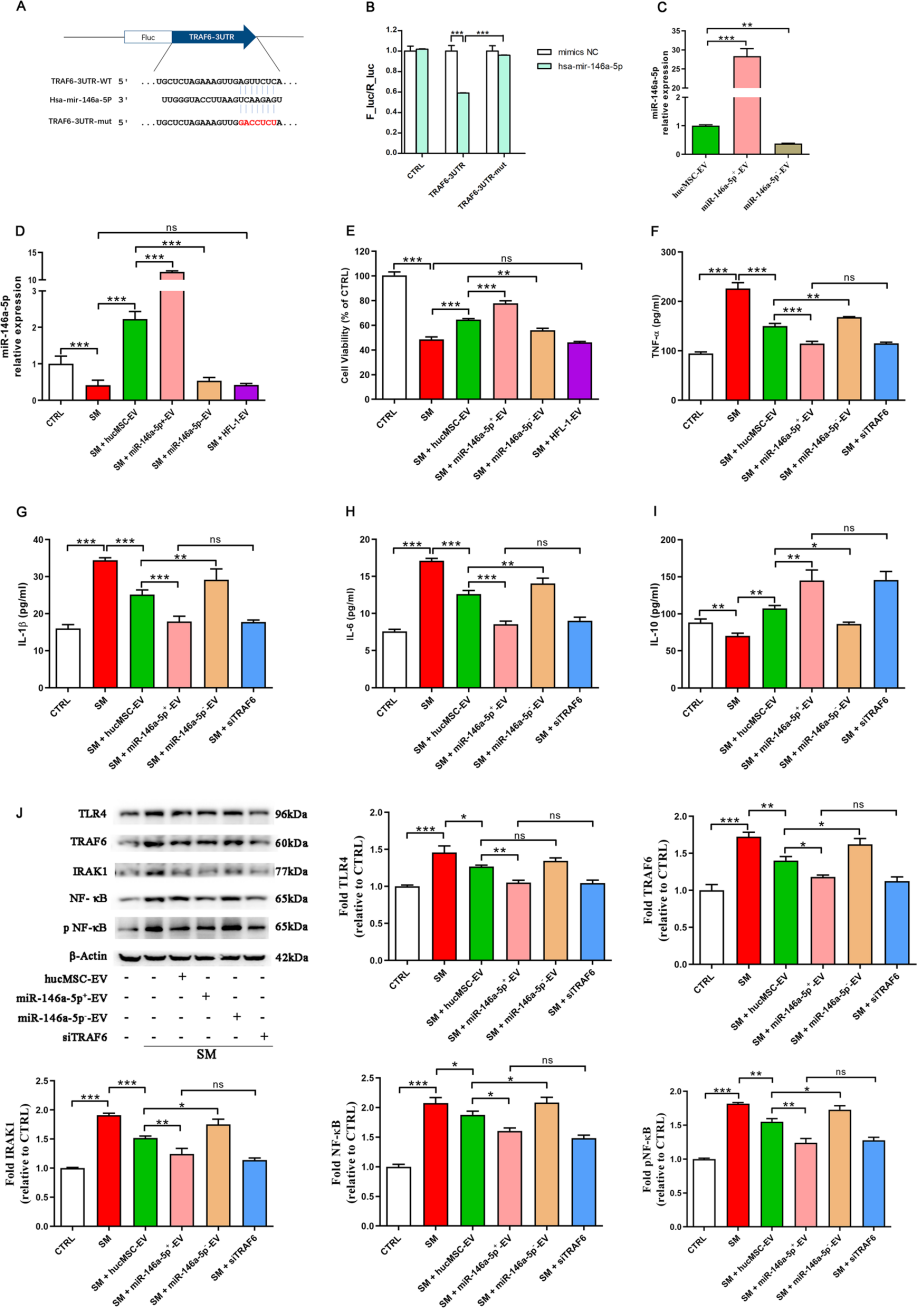
MiR-146a-5p delivered by hucMSC-EVs targets TRAF6 and mediates the anti-inflammatory effect of hucMSC-EVs in vitro. **A** Potential miR-146a-5p binding site on the 3′-UTR of TRAF6. **B** The luciferase assay shows that the cotransfection of the TRAF6 3′-UTR and miR-146a-5p mimic reduced luciferase activity in HEK293 cells. **C** Relative levels of miR-146a-5p in extracellular vesicles derived from hucMSCs transfected with the miR-146a-5p mimic or inhibitor. **D** miR-146a-5p expression in RAW264.7 cells from each group was assessed by qRT–PCR. **E** The cell viability of different treatment groups was determined by the CCK-8 assay. **F**–**I** The levels of TNF-α, IL-1β, IL-6, and IL-10 in the cell supernatant isolated at 24 h after exposure to SM were visualized by ELISA. **J** The expression of TLR4/NF-κB signaling pathway-related proteins isolated from cells at 24 h after exposure to SM was visualized by western blotting. Full-length blots are presented in Additional file 1: Fig. S6. The data are presented as the mean ± SD of individuals included in each group (*n* = 3) and representative of at least three independent experiments. *P < 0.05; **P < 0.01; and ***P < 0.001

We examined potential mediators of the effect exhibited by miR-146a-5p delivered by hucMSC-EVs. MiR-146a-5p could bind to the TRAF6 mRNA 3′-untranslated region (3′-UTR), which was confirmed by sequence alignment with TargetScan software (Fig. 4A). The combination of seven complementary nucleotides was altered to construct a mutated version of the TRAF6 3′-UTR. This mutated TRAF6 3′-UTR was added to the luciferase coding region and cotransfected into HEK293T cells with miR-146a-5p mimic. The luciferase activity was significantly lower when the TRAF6 3′-UTR was cotransfected with miR-146a-5p mimic than that cotransfected with the control. However, it was obvious that the mutant TRAF6 3′-UTR reduced this effect (Fig. 4B). These results confirmed that miR-146a-5p specifically targeted and suppressed the 3′-UTR of TRAF6.

To explore the mechanism of suppressed inflammation by miR-146a-5p in hucMSC-EVs, miR-146a-5p-overexpressing extracellular vesicles (miR-146a-5p^+^-EVs) or miR-146a-5p-underexpressing extracellular vesicles (miR-146a-5p^−^-EVs) were isolated from hucMSCs transfected with miR-146a-5p mimic or inhibitor, respectively (Fig. 4C). We treated RAW264.7 cells with extracellular vesicles or miR-146a-5p^+^/miR-146a-5p^−^-EVs after SM injury. The expression of miR-146a-5p (Fig. 4D) and the cell viability (Fig. 4E) were increased after hucMSC-EVs treatment and were significantly increased or reduced in the miR-146a-5p^+^-EV group or miR-146a-5p^−^-EV group in comparison with the hucMSC-EV group, respectively. However, comparing the HFL-1-EV group with the SM group, no significant difference was found. The same results were detected in lung epithelial cells (Additional file 1: Fig. S3A, B). These results suggested that the alleviation of SM-induced cytotoxicity and promotion of recovery in RAW264.7 cells by hucMSC-EVs were mediated through miR-146a-5p delivered by hucMSC-EVs.

**Fig. 5:**
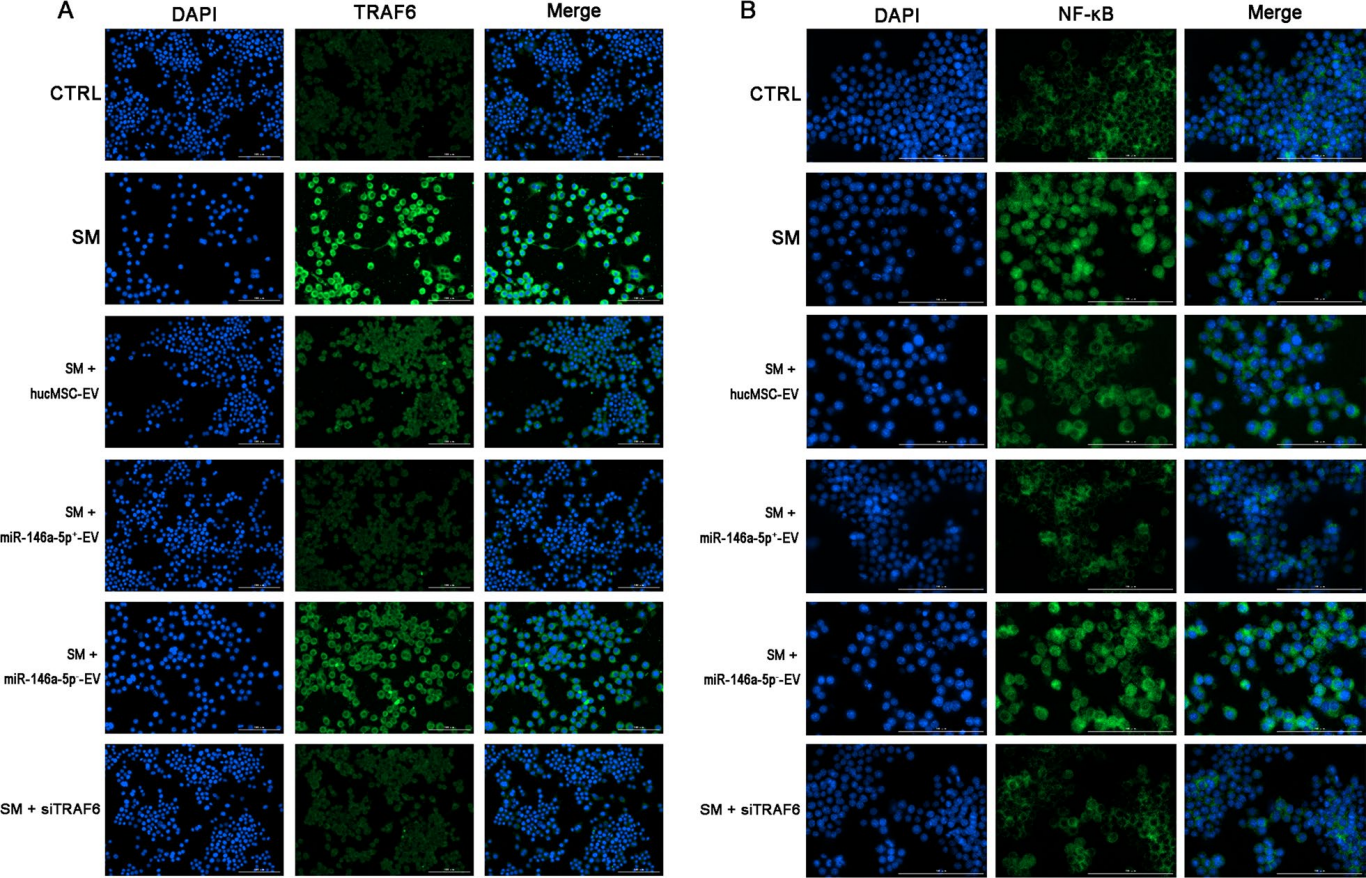
Representative immunofluorescence images of TRAF6 (**A** × 200) and NF-κB (**B** × 400) expression in RAW264.7 cells (× 200) (*n* = 3 mice/group). Scale bar = 100 μm

To identify whether the abovementioned effect of miR-146a-5p is TRAF6 dependent, RAW264.7 cells were treated with hucMSC-EVs, miR-146a-5p^+^-EVs, or miR-146a-5p^−^-EVs or were transfected with siTRAF6. The ELISA results showed that miR-146a-5p^+^-EVs administration successfully diminished the secretion of TNF-α (Fig. 4F), IL-1β (Fig. 4G), and IL-6 (Fig. 4H) in RAW264.7 cells after SM exposure. The level of the anti-inflammatory factor IL-10 (Fig. 4I) was significantly augmented in the miR-146a-5p^+^-EVs group. The effect of miR-146a-5p^+^-EVs was the same as transfection with siTRAF6 but was more obvious than that of hucMSC-EVs administration. MiR-146a-5p^−^-EVs aggravated the inflammatory response, as opposed to miR-146a-5p^+^-EVs. These data suggested that miR-146a-5p delivered by hucMSC-EVs mediated the anti-inflammatory effect of hucMSC-EVs. We further explored the mechanism. As shown in Fig. 4J, TLR4, TRAF6, IRAK1, NF-κB, and pNF-κB protein expression was reduced after hucMSC-EVs treatment and was further significantly downregulated in the miR-146a-5p^+^-EVs group compared to hucMSC-EVs administration. In addition, comparing the miR-146a-5p^+^-EV group with the siTRAF6 group, no significant difference was found. The same results have been shown in lung epithelial cells (Additional file 1: Fig. S3C). To observe the influence of extracellular vesicles and siRNA on TRAF6 (Fig. 5A) and NF-κB (Fig. 5B) expression, we detected these proteins in RAW264.7 cells by immunofluorescent staining after multiple treatments. MiR-146a-5p^+^-EV administration significantly downregulated TRAF6 expression and NF-κB nuclear translocation compared to hucMSC-EVs administration, and the effect was the same as that of the siTRAF6 group. MiR-146a-5p^−^-EVs showed the opposite effect relative to miR-146a-5p^+^-EVs. The above results demonstrated that miR-146a-5p delivered by hucMSC-EVs targeted TRAF6 and mediated the anti-inflammatory effect of hucMSC-EVs in vitro.

### MiR-146a-5p delivered by hucMSC-EVs is involved in the hucMSC-EV-mediated therapeutic effects in vivo

**Fig. 6:**
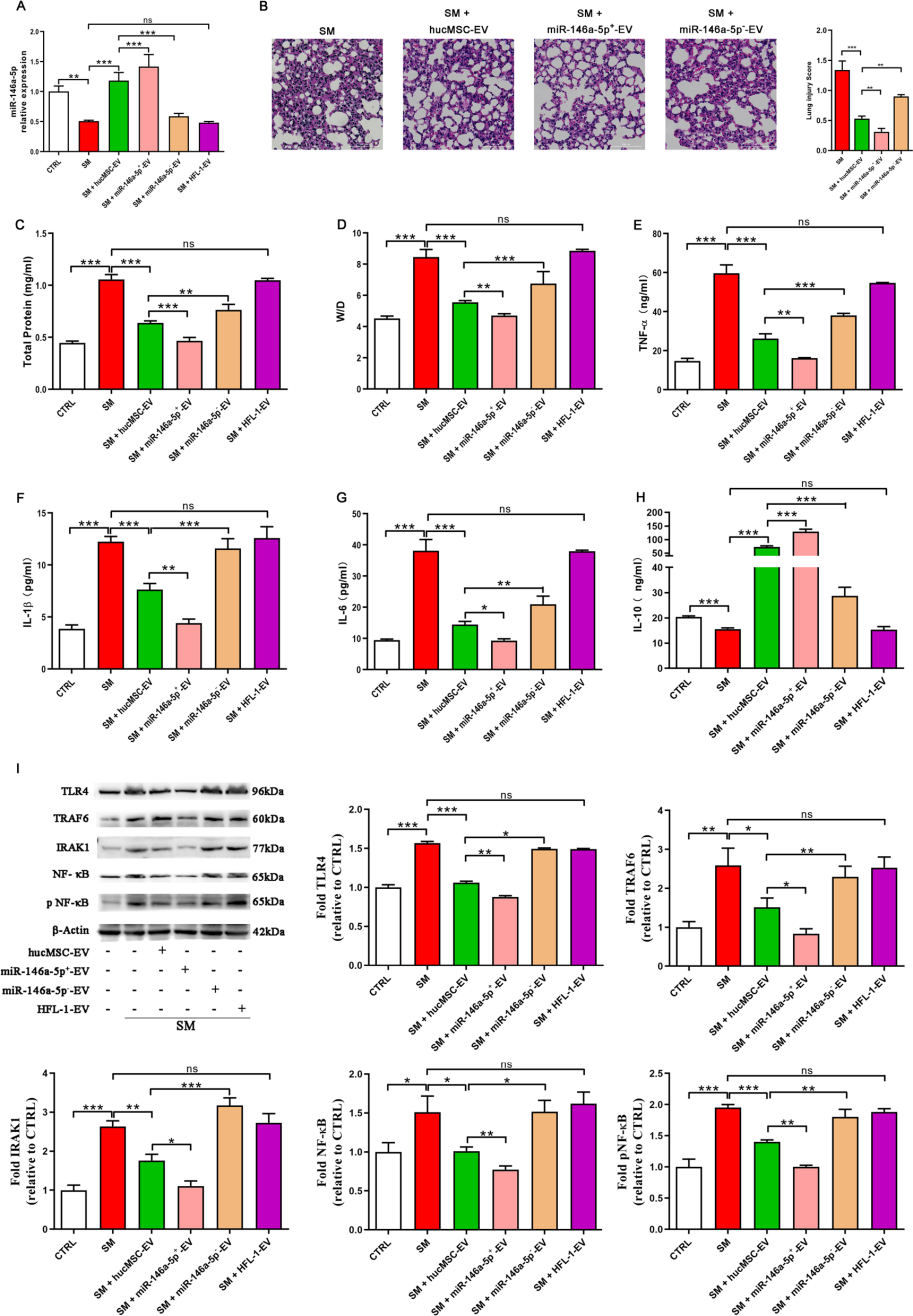
MiR-146a-5p delivered by hucMSC-EVs is involved in the hucMSC-EV-mediated therapeutic effects in vivo. ICR mice were subcutaneously injected with diluted SM (30 mg/kg weight). Extracellular vesicles (6 × 10^8^ particles) were injected into the mice through the tail vein on the first and third days after SM exposure. On the fifth day, the lung tissues were collected for subsequent experiments. **A** miR-146a-5p expression in mouse lung tissues was examined by qRT–PCR (*n* = 6 mice/group). **B** Representative histological micrograph analysis (× 200, scale bar = 100 μm) and histopathological scores (*n* = 3 mice/group). **C**, **D** Comparison of BALF protein and the wet/dry weight ratio in SM-exposed mice (*n* = 6 mice/group). **E**–**H** ELISA showing the expression of the inflammatory factors TNF-α, IL-1β, IL-6, and IL-10 in mouse lung tissues (*n* = 6 mice/group). **I** The expression of TLR4/NF-κB signaling pathway-related proteins isolated from lung tissues was visualized by western blotting (*n* = 3 mice/group). Full-length blots are presented in Additional file 1: Fig. S7. The data are presented as the mean ± SD of individuals included in each group and representative of at least three independent experiments. *P < 0.05; **P < 0.01; and ***P < 0.001

To explore the mechanism of the therapeutic effects of hucMSC-EVs on SM injury in vivo, the mice were treated with different types of extracellular vesicles via tail vein injection on the first and third days after SM exposure. Compared with the therapeutic effects of miR-146a-5p^+^-EVs, miR-146a-5p^−^-EVs, and hucMSC-EVs, we found that miR-146a-5p was significantly increased or reduced in the miR-146a-5p^+^-EV group or the miR-146a-5p^−^-EV group compared with the hucMSC-EV group, respectively (Fig. 6A). MiR-146a-5p^+^-EVs dramatically decreased ALI induced by SM, as shown by H&E staining (Fig. 6B), BALF protein (Fig. 6C), the wet/dry weight ratio (Fig. 6D), and the expression of Ki67 (Additional file 1: Fig. S4), compared to those of the hucMSC-EV group. The proinflammatory factors present in mouse lung tissues and serum samples were examined using ELISA. Compared with the levels in the hucMSC-EV group, the levels of TNF-α (Fig. 6E), IL-1β (Fig. 6F), and IL-6 (Fig. 6G) in the miR-146a-5p^+^-EV group were markedly reduced, while the level of the anti-inflammatory factor IL-10 (Fig. 6H) was significantly increased. MiR-146a-5^−^-EVs showed the opposite effect compared to that of miR-146a-5p^+^-EVs. These results suggested that miR-146a-5p delivered by hucMSC-EVs mediated the anti-inflammatory effect of hucMSC-EVs, which was the same as the results of in vitro experiments.

**Fig. 7:**
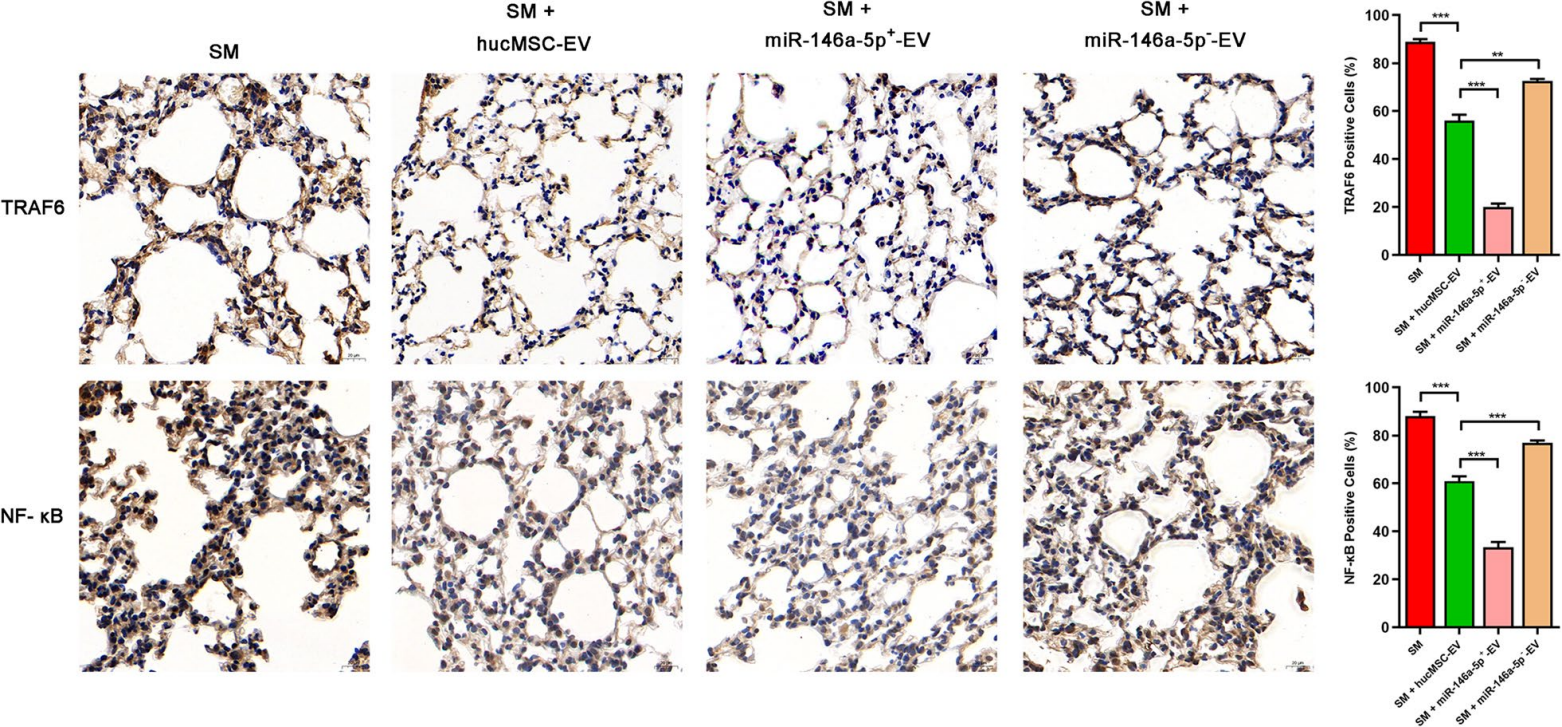
Representative immunohistochemistry images of TRAF6 and NF-κB expression in tissues (× 400) (*n* = 3 mice/group). Scale bar = 20 μm, *P < 0.05; **P < 0.01; and ***P < 0.001

The mechanism was further explored. As shown in Fig. 6I, TLR4, TRAF6, IRAK1, NF-κB, and pNF-κB protein expression was significantly downregulated in the miR-146a-5p^+^-EV group compared to the hucMSC-EV group. The influence of different types of extracellular vesicles on TRAF6 and NF-κB expression was evaluated by immunohistochemical staining (Fig. 7). MiR-146a-5p^+^-EV administration significantly downregulated TRAF6 expression and NF-κB nuclear translocation compared to hucMSC-EVs administration. MiR-146a-5p^−^-EVs showed the opposite effect to that of miR-146a-5p^+^-EVs.

These results were also the same as the in vitro results described above, suggesting that miR-146a-5p delivered by hucMSC-EVs targeted TRAF6 and mediated the anti-inflammatory effect of hucMSC-EVs in vivo.

## Discussion

In the current research, we found a therapeutic effect of hucMSC-EVs on ALI caused by SM, especially in terms of improving the inflammatory response. Treatment with hucMSC-EVs increased the survival rate, attenuated ALI, reduced BALF protein and the wet/dry weight ratio, and suppressed proinflammatory cytokine levels. Through an analysis of the function and contents of extracellular vesicles, the delivery of bioactive miRNAs by extracellular vesicles to cells is regarded as the potential mechanism of these effects. We found that the miR-146a-5p expression levels markedly decreased after SM injury, and their expression could be augmented in lung tissue or cells by treatment with hucMSC-EVs. Moreover, by transfecting miR-146a-5p mimic or inhibitor to enhance or mitigate its expression in RAW264.7 cells, respectively, we demonstrated that miR-146a-5p alleviated SM-induced cytotoxicity and significantly suppressed inflammation by regulating the TLR4 signaling pathway. Finally, miR-146a-5p^+^/miR-146a-5p^−^-EVs were isolated and collected by modifying hucMSCs. We discovered that miR-146a-5p^+^-EVs administration significantly attenuated TRAF6 expression, which negatively regulated SM-induced inflammation in vitro and in vivo. Taken together, these accumulated data indicate that hucMSC-EVs could prevent inflammation and improve ALI induced by SM through the delivery of miR-146a-5p.

In recent years, the potential role of stem cells as a therapy has attracted considerable attention. MSCs are non-hematopoietic cells which can be isolated from various sources, including umbilical cord, adipose tissue, bone marrow, and human dental pulp [33, 34]. The therapeutic efficacy of mouse BMSCs and their extracellular vesicles in SM-induced lung injury has been reported by our group [14, 15]. Due to limited tissue sources, immunogenicity, and ethical restrictions on studying BMSCs, we explored the utility of hucMSCs in treating SM-ALI. HucMSC surface antigens are not prominent, the rejection reactions of transplanted cells are insignificant, and the matching requirements are not strict, which makes them convenient for use in allotransplantation. Numerous studies have confirmed that hucMSCs possess potential effects in treating pulmonary disease including COVID-19 [12, 35]. In addition, positive outcomes from hucMSCs in treating diseases have been found in several clinical trials evaluating their therapeutic efficacy [35–37]. It has been suggested that MSCs derived from bone marrow are potentially good candidates for brain and spinal cord injury treatment, MSCs derived from adipose tissue are potentially good candidates for reproductive disorder treatment and skin regeneration, MSCs derived from the umbilical cord are potentially good candidates for pulmonary disease and acute respiratory distress syndrome treatment [38]. However, the clinical application of hucMSCs is limited because of their differentiation and proliferation abilities, which are related to long-term culture and donor age [39]. Therefore, we focused on hucMSC-EVs as an alternative strategy that could have the same therapeutic ability as hucMSCs while eliminating the complications of cell transplantation [40]. In our study, the therapeutic effect of hucMSCs-EVs was evaluated by injecting extracellular vesicles into the tail veins of mice with ALI caused by SM. Compared with the control groups, we discovered that extracellular vesicles derived from hucMSCs protected against SM-induced ALI by attenuating inflammation.

HucMSCs-EVs are natural materials and serve as a vehicle that could alter the gene expression of the recipient cells by transferring protein, mRNA, and miRNA to distant recipient cells [41, 42]. Recent reports have suggested that extracellular vesicles have diverse potential applications in therapeutics by delivering miRNAs and could be modified by manipulating hucMSCs [43]. In the nucleus of hucMSCs, a primary transcript (pri-miRNA) is transcribed from miRNA genes, and it is processed into a precursor molecule (pre-miRNA). The pre-miRNA will then appear in the cytoplasm, which is transported by the transport protein. In the cytoplasm, mature miRNA is further processed by pre-miRNA. These mature miRNAs could be loaded in the multivesicular bodies produced by invagination of the early endosomal membrane. These vesicles dock on the cell membrane, following which the positive extracellular vesicles are released and present in the extracellular space. Extracellular vesicles fuse with the recipient cell membrane or are phagocytosed, followed by membrane fusion. MiRNAs in extracellular vesicles are released into the cytoplasm of recipient cells to produce translational repression. Wei et al. confirmed that exosomal miR-377-3p played an ameliorative role by inducing autophagy in in vitro and in vivo models of lipopolysaccharide-induced ALI [44]. A previous study found that hucMSCs-EVs modulated miR-451 to improve inflammation and thus restore tissue health after burn-induced ALI [45]. MiR-181c released by hucMSCs-EVs mitigated burn-induced inflammation, as reported by Li et al. [46]. In our study, extracellular vesicles from hucMSCs improved the outcomes of SM-induced ALI through the delivery of miR-146a-5p.

A body of evidence indicates that microRNAs play important roles in suppressing posttranscriptional genes expression. MicroRNAs play important roles in many pathophysiological processes. Many external and internal stimulating factors can cause inflammation. These non-self antigens can trigger both innate and adaptive immune responses. MiR-146a-5p, a small noncoding RNA molecule, is an important regulator of the innate immune response and is closely related to inflammation [47–49]. Relevant proteins in the canonical NF-κB pathway, such as TLR-4, MyD88, IRAK1, and TRAF6, can be downregulated by miR-146a-5p. MiR-146a-5p is a functional biomarker of inflammation because of its ability to modulate the NF-κB pathway. Increasing evidence suggests that miR-146a-5p, which binds to various downstream targets including TRAF6 [50], plays a role in improving inflammation in many biological processes [51–54]. Through selective targeting of TRAF6 proteins by miR-146a-5p, LPS-induced inflammatory responses can be inhibited in endothelial cells. Wang et al. reported that miR-146a exerts a protective role in myocardial injury, which was related to preventing the production of inflammatory cytokines by miR-146a inhibiting the expression of TRAF6 [55]. However, the function of miR-146a-5p delivered by hucMSC-EVs in SM-induced lung injury were unclear. In our experiments, we proved the miR-146a-5p mimic could down-regulate TRAF6 in SM-induced ALI, then we found that miR-146a-5p-overexpressing extracellular vesicles (miR-146a-5p^+^-EVs) has the similar effects, which is similar to the effects of siTRAF6. These results demonstrated that miR-146a-5p delivered by hucMSC-EVs targeted TRAF6 and mitigated inflammation induced by SM.

ALI is an overactivated inflammation caused by direct or indirect injuries, which can destroy lung parenchymal cells and significantly reduce lung function. We established the mouse model of ALI by sulfur mustard. After sulfur mustard injury, immune cells will be activated, and the activated immune cells will release excessive proinflammatory factors, chemokines and proteases, resulting in the damage of lung parenchymal cells such as vascular endothelial cells and alveolar epithelial cells [56, 57]. Since inflammation plays an important role in the pathogenesis of ALI, we are more concerned about the effect and mechanism of extracellular vesicles on the inflammation of SM-induced ALI. Due to heterogeneity and etiology, the pathogenesis of ALI remains unclear. With the progress of research, several inflammation-related mechanisms in ALI have been discovered. In addition to the TLRs mediate NF-κB signaling pathway involved in our study, there are also JAK2/STAT3, NLRP3 inflammasome, PI3K/AKT, p38 MAPK and so on [58]. Whether there are other miR-146a-5p related pathways or other components involved in the hucMSC-EV-induced improvement in ALI caused by SM remains to be further studied because the components of extracellular vesicles are very diverse, and the injury mechanism of SM is complicated and has not been clearly elaborated. We will continue to perform related studies in the future to explain the mechanism of hucMSC-EVs in improving lung injury caused by SM.

## Conclusion

**Fig. 8:**
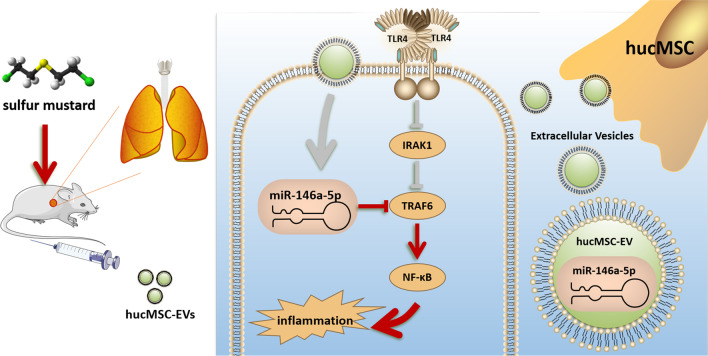
MiR-146a-5p delivered by hucMSC-EVs targeted TRAF6, causing hucMSC-EVs to exert anti-inflammatory effects in SM-induced ALI. This figure was modified from Servier Medical Art (http://smart.servier.com/), licensed under a Creative Commons Attribution 3.0 Unported License (https://creativecommons.org/licenses/by/3.0/)

We demonstrated that hucMSC-EVs can effectively ameliorate SM-induced inflammation and ALI. Further studies demonstrated the importance of miR-146a-5p in the regulation of inflammation by hucMSC-EVs (Fig. 8). Thus, we elucidated the key role of miR-146a-5p delivered by hucMSC-EVs in the amelioration of SM-induced inflammation, which might provide a therapeutic approach for the clinical rescue of SM victims.

## Supplementary Information

** Additional file 1. **Supplementary experimental procedures, figures, and full-length blots.

## Data Availability

The datasets used and/or analysed during the current study are available from the corresponding author on reasonable request.

## Abbreviations

SM: Sulfur mustard
ALI: Acute lung injury
ACWs: Abandoned chemical weapons
HucMSC-EVs: Human umbilical cord mesenchymal stromal cell extracellular vesicles
MSC: Mesenchymal stem cell
DMEM: Dulbecco's modified Eagle medium
α-MEM: Alpha minimum essential medium
FBS: Fetal bovine serum
PBS: Phosphate buffered saline
NAC: *N*-Acetyl cysteine
BALF: Bronchoalveolar lavage fluid
BCA: Bicinchoninic acid assay
CCK-8: Cell counting kit-8
ELISA: Enzyme-linked immunosorbent assay
TNF-α: Tumor necrosis factor-α
TLR4: Toll-like receptor 4
TRAF6: TNF receptor associated factor 6
IRAK1: Interleukin-1 receptor-associated kinase 1
NF-κB: Nuclear factor kappa-B
