# Supplementary material for: MiR-146a-5p delivered by hucMSC extracellular vesicles modulates the inflammatory response to sulfur mustard-induced acute lung injury
Source: Stem Cell Res Ther. 2023 May 30;14:149. doi: 10.1186/s13287-023-03375-8 (PMC10227795; doi:10.1186/s13287-023-03375-8)
Supplement: Supplementary file 1 — Additional file 1. Supplementary experimental procedures, figures, and full-length blots. [file 13287_2023_3375_MOESM1_ESM.docx]

## Characterization of human umbilical cord MSCs

The hucMSC markers were determined by flow cytometry analyses. The results showed that the cells stained positive for CD29 (99.83%), CD44 (99.89%), CD73 (99.78%), CD105 (99.42%) and CD166 (96.36%) and stained negative for CD11b (0.18%), CD34 (0.54%) and CD45 (0.19%). The adipogenesis, osteogenesis, and chondrogenesis potential of the cells were confirmed by red oil O staining, alizarin red staining, and alcian blue staining, respectively (Fig. S1).


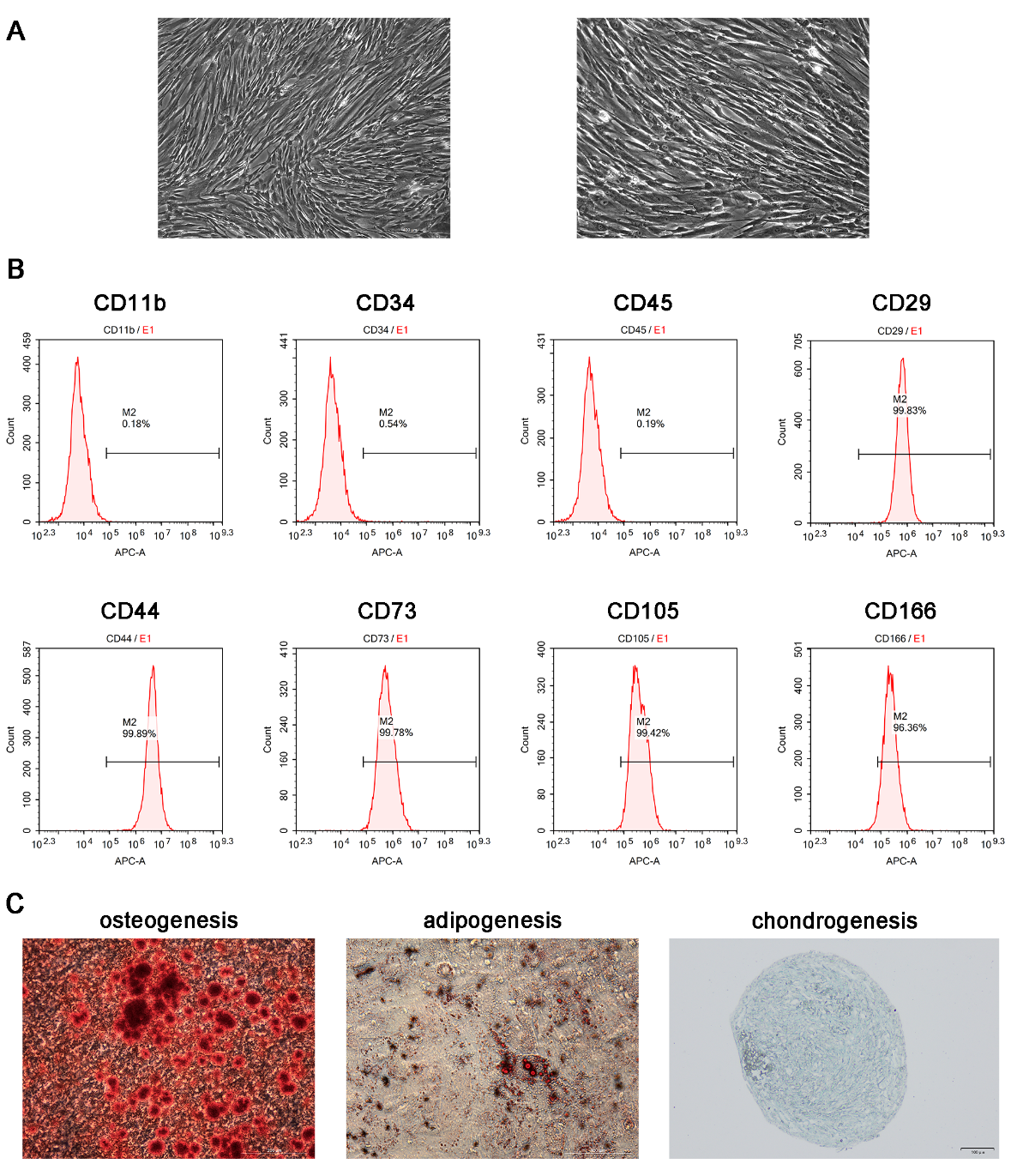


**Fig. S1** **Characterization of human umbilical cord MSCs (****hucMSCs)**

(A) Morphology of hucMSCs (× 50 on the left and × 100 on the right). (B) Immune-phenotype of hucMSCs positive for CD29, CD44, CD73, CD105 and CD166 and negative for CD11b, CD34 and CD45. (C) Oil red O staining of adipo-induced hucMSCs, alizarin red staining of osteo-induced hucMSCs and alcian blue staining of chondro-induced hucMSCs.

## Characterization of extracellular vesicles derived from hucMSCs

As shown in Fig. S2, the identity and purity of the extracellular vesicles were characterized by several methods, such as nanoparticle tracking analysis, transmission electron microscopy, and western blotting. The nanoparticles exhibited a typical round-cup structure by transmission electron microscopy. Nanoparticle tracking analysis recorded the nanoparticle size distribution, and the nanoparticles had a typical modal size of 40-1000 nm (Fig. S2B). Western blotting showed that the exosomal surface markers CD9, CD63, CD81, and Cav-1 were enriched in these nanoparticles (Fig. S2C). The nanoparticles labeled with Dil fluorescent dye were incubated with the cells. Confocal microscopy showed that the nanoparticles could be ingested by the target cells (Fig. S2D). Thus, the results suggested that these nanoparticles were extracellular vesicles and could be used in subsequent experiments.


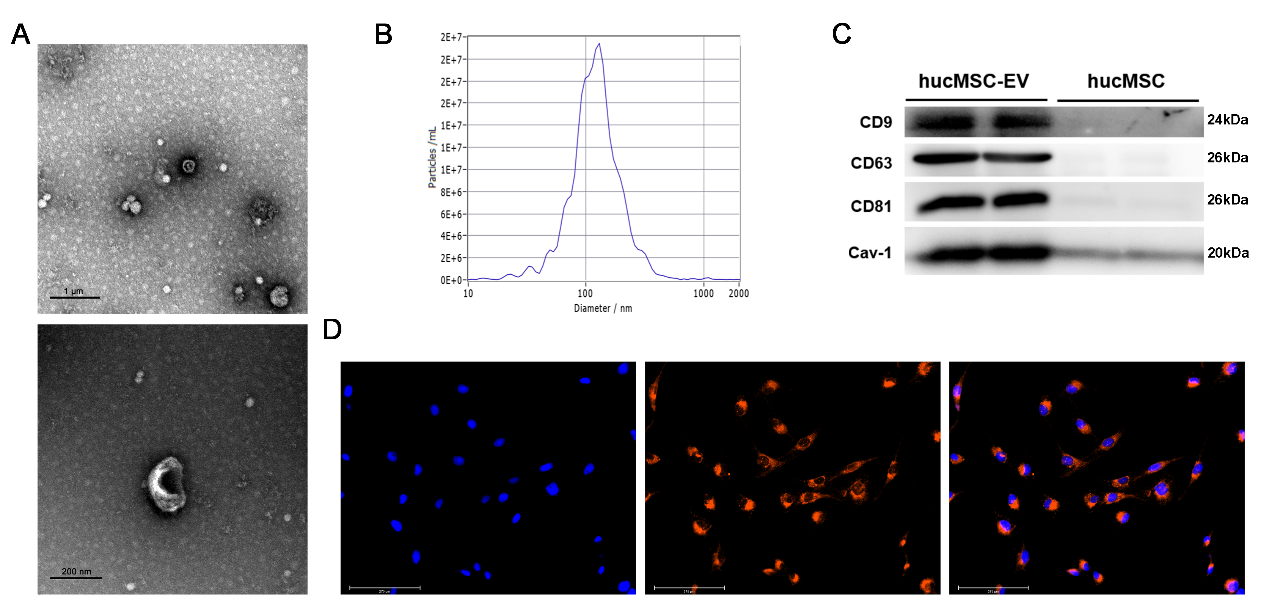


**Fig. S2 Characterization of** **extracellular vesicles derived from hucMSCs.**

(A) Identification of a representative image of hucMSC-EVs by transmission electron microscopy. (B) Nanoparticle tracking analysis recorded the hucMSC-EV diameter range. (C) The expression of the exosomal surface markers CD9, CD63, CD81 and Cav-1 was determined by western blotting. (D) HucMSC-EVs labeled with Dil fluorescent dye were incubated with BEAS-2B cells. Confocal microscopy showed that hucMSC-EVs could be ingested by the target cells. Scale bar= 275 μm.

## MiR-146a-5p delivered by hucMSC-EVs targets TRAF6 and mediates the anti-inflammatory effect of hucMSC-EVs

We treated BEAS-2B cells with extracellular vesicles or miR-146a-5p^+^/ miR-146a-5p ^-^-EVs after SM injury. The expression of miR-146a-5p (Fig. S3A) and the cell viability (Fig. S3B) were increased after hucMSC-EVs treatment and were significantly increased or reduced in the miR-146a-5p^+^-EV group or miR-146a-5p^-^-EV group in comparison with the hucMSC-EV group, respectively. However, comparing the HFL-1-EV group with the SM group, no significant difference was found. These results suggested that the alleviation of SM-induced cytotoxicity and promotion of recovery in BEAS-2B cells by hucMSC-EVs were mediated through miR-146a-5p delivered by hucMSC-EVs. We further explored the mechanism. As shown in Fig. S3C, TLR4, TRAF6, IRAK1, NF-κB, and pNF-κB protein expression was reduced after hucMSC-EVs treatment and was further significantly downregulated in the miR-146a-5p^+^-EVs group compared to hucMSC-EVs administration. In addition, comparing the miR-146a-5p^+^-EV group with the siTRAF6 group, no significant difference was found. The above results demonstrated that miR-146a-5p delivered by hucMSC-EVs targeted TRAF6 and mediated the anti-inflammatory effect of hucMSC-EVs. To explore the therapeutic effects of hucMSC-EVs on SM injury in vivo, the mice were treated with different types of extracellular vesicles via tail vein injection on the first and third days after SM exposure. The influence of different types of extracellular vesicles on the expression of Ki67 was evaluated by immunohistochemical staining (Supplementary Fig. S4). MiR-146a-5p^+^-EV administration significantly upregulated the expression of Ki67 compared to hucMSC-EVs administration. MiR-146a-5p^-^-EVs showed the opposite effect to that of miR-146a-5p^+^-EVs.


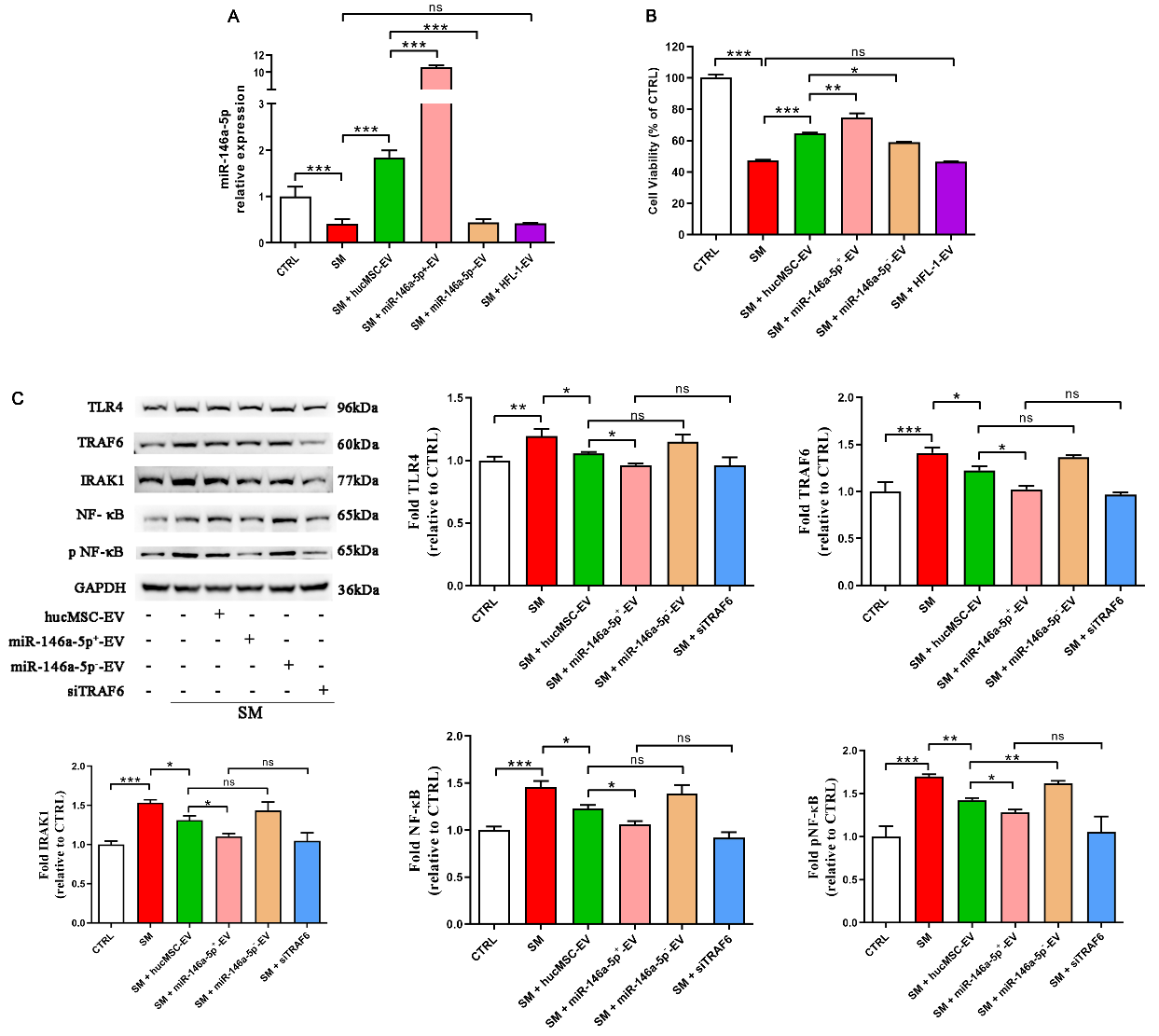


**Fig. S3** **MiR-146a-5p delivered by hucMSC-EVs targets TRAF6 and mediates the anti-inflammatory effect of hucMSC-EVs in** **lung epithelial cell.**

(A) miR-146a-5p expression in BEAS-2B cells from each group was assessed by qRT–PCR. (B) The cell viability of different treatment groups was determined by the CCK-8 assay. (C) The expression of TLR4/NF-κB signaling pathway-related proteins isolated from BEAS-2B cells at 24 h after exposure to SM was visualized by western blotting. Full-length blots are presented in Supplementary Fig. S8. The data are presented as the mean ± SD of individuals included in each group (n=3) and representative of at least three independent experiments. **P* < 0.05; ***P* < 0.01; and ****P* < 0.001.


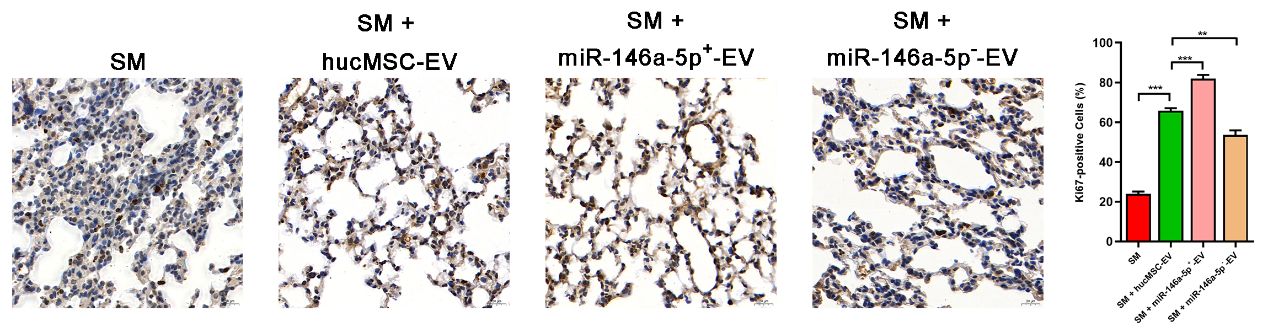


**Fig. S4 Representative immunohistochemistry images of Ki67 expression in tissues (×400) (n=3 mice/group). Scale bar= 20 μm.** ***P* < 0.01; and ****P* < 0.001.

**
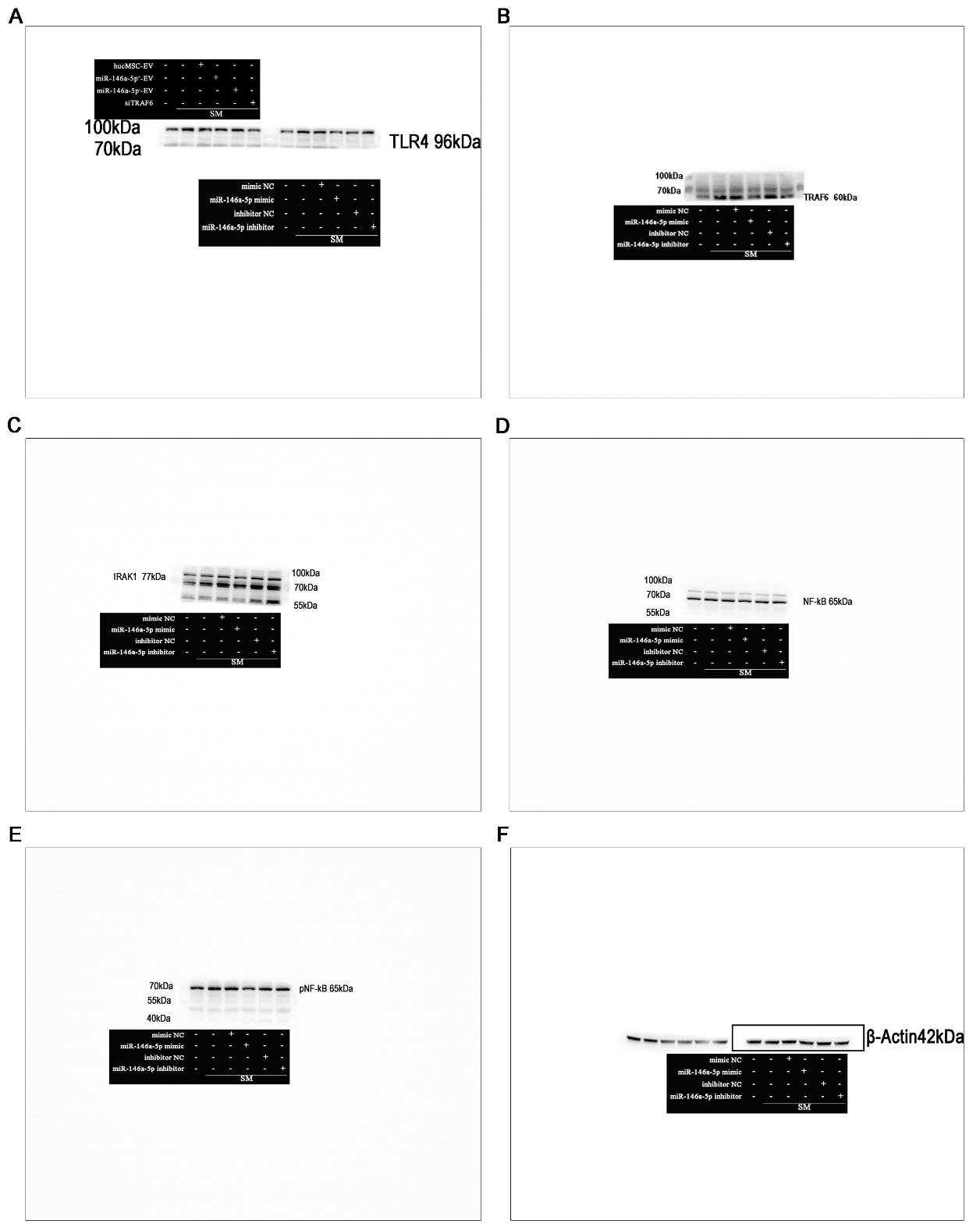
**

**Fig. S5 Full-length blots of Fig. 3J.**

(A) TLR4 (right). (B) TRAF6. (C) IRAK1. (D) NF-κB. (E) pNF-κB. (F) β-Actin.


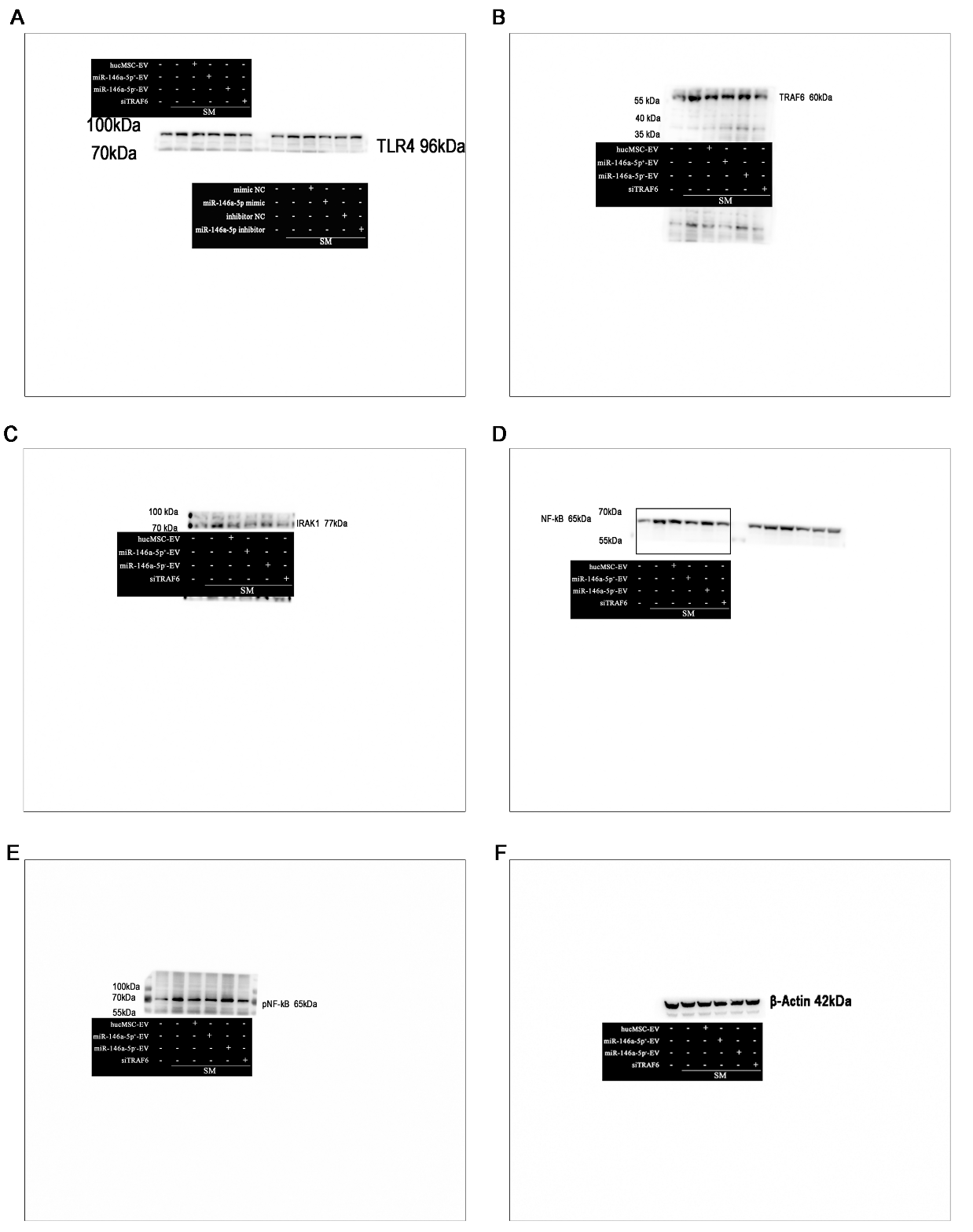


**Fig. S6 Full-length blots of Fig. 4J.**

(A) TLR4 (left). (B) TRAF6. (C) IRAK1. (D) NF-κB. (E) pNF-κB. (F) β-Actin.


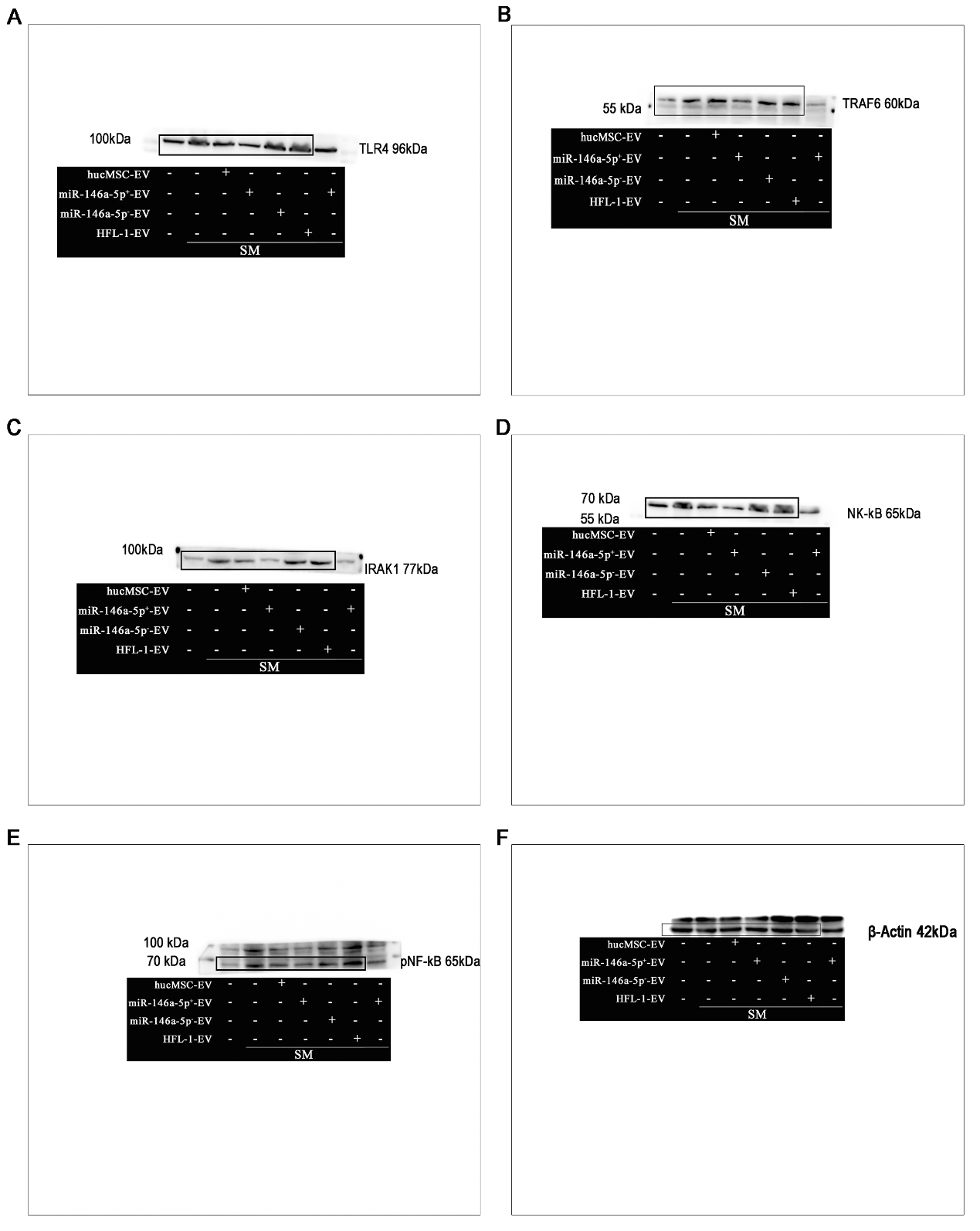


**Fig. S7 Full-length blots of Fig. 6I.**

(A) TLR4. (B) TRAF6. (C) IRAK1. (D) NF-κB. (E) pNF-κB. (F) β-Actin.


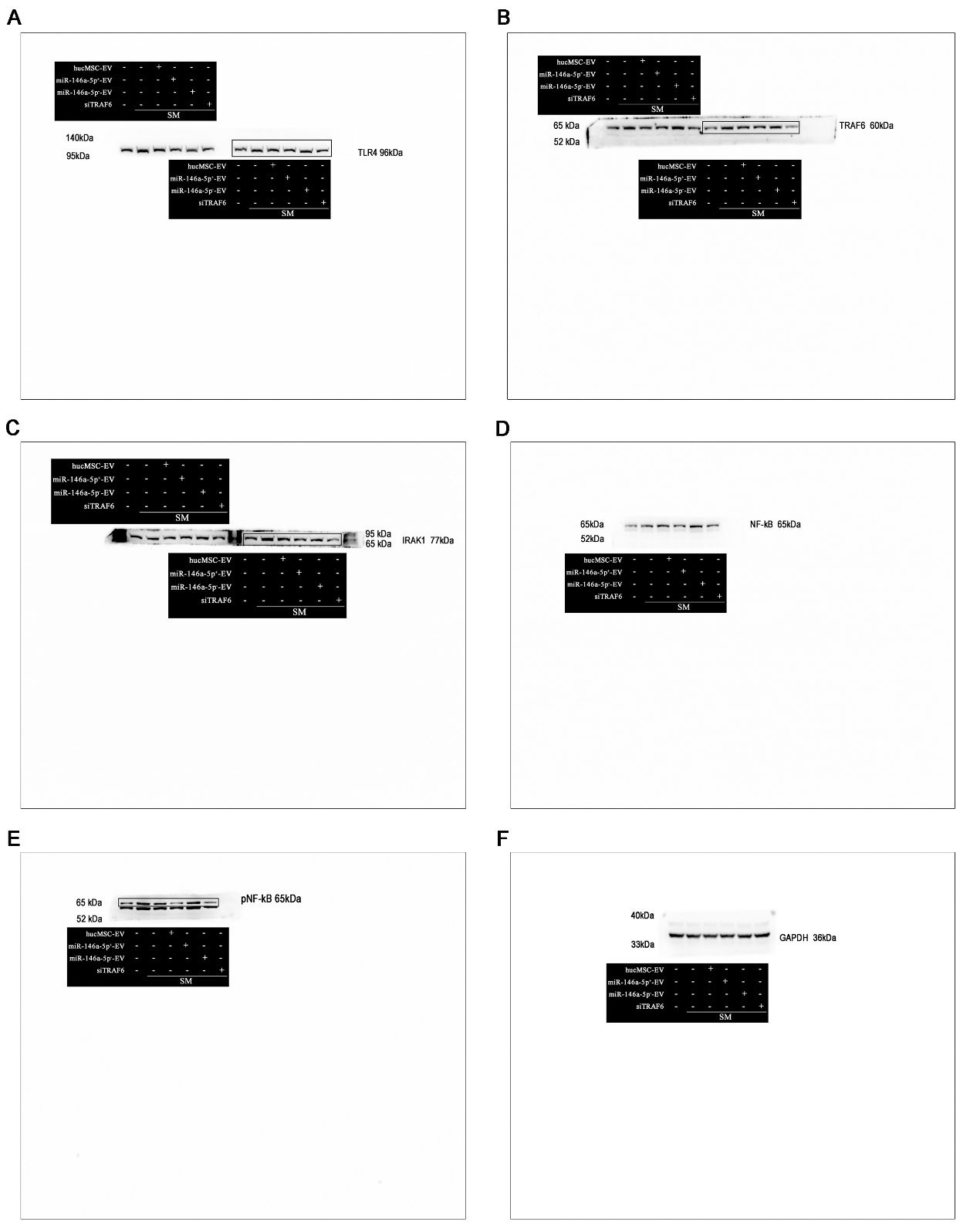


**Fig. S8 Full-length blots of Fig. S3C.**

(A) TLR4 (right). (B) TRAF6 (right). (C) IRAK1 (right). (D) NF-κB. (E) pNF-κB. (F) GAPDH.
